# Supplementary material for: Conditional chemoconnectomics (cCCTomics) as a strategy for efficient and conditional targeting of chemical transmission
Source: eLife. 2024 Apr 30;12:RP91927. doi: 10.7554/eLife.91927 (PMC11060718; doi:10.7554/eLife.91927)
Supplement: Figure 3—source data 3. [file elife-91927-fig3-data3.zip › Figure 3 source data3 part 1.zip!/Figure 3-Source data 3-part1/1071f2-1-CRISPResso_Report_ztwktvfp.zip!/CRISPResso_on_1071f2_4/9.Alleles_frequency_table_around_sgRNA_gcagaaggatttgtaaatgg.pdf]

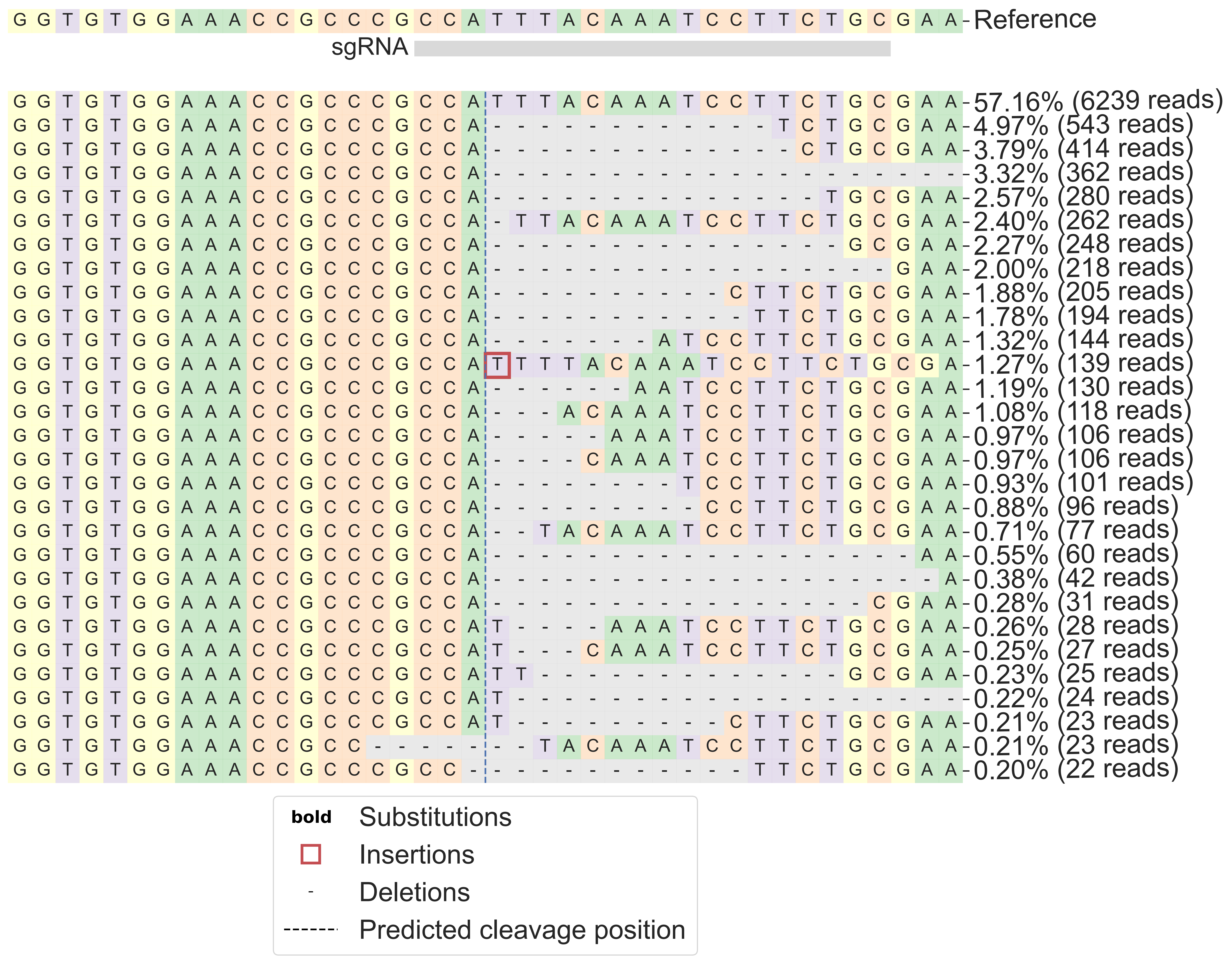

Reference
sgRNA
57.16% (6239 reads)
4.97% (543 reads)
3.79% (414 reads)
3.32% (362 reads)
2.57% (280 reads)
2.40% (262 reads)
2.27% (248 reads)
2.00% (218 reads)
1.88% (205 reads)
1.78% (194 reads)
1.32% (144 reads)
1.27% (139 reads)
1.19% (130 reads)
1.08% (118 reads)
0.97% (106 reads)
0.97% (106 reads)
0.93% (101 reads)
0.88% (96 reads)
0.71% (77 reads)
0.55% (60 reads)
0.38% (42 reads)
0.28% (31 reads)
0.26% (28 reads)
0.25% (27 reads)
0.23% (25 reads)
0.22% (24 reads)
0.21% (23 reads)
0.21% (23 reads)
0.20% (22 reads)
bold Substitutions
Insertions
Deletions
Predicted cleavage position
